# A Case Report of Distal Femur Fungal Osteomyelitis

**DOI:** 10.7759/cureus.109316

**Published:** 2026-05-20

**Authors:** Vinith M, Mohammed Tavfiq, Giriraj Harshavardhan, Sundar Suriyakumar

**Affiliations:** 1 Department of Orthopaedics, Sri Ramachandra Institute of Higher Education and Research, Chennai, IND

**Keywords:** candida auris, case report, distal femur, fungal osteomyelitis, voriconazole

## Abstract

Fungal osteomyelitis is an uncommon infection that can present with nonspecific symptoms, leading to delays in diagnosis. We report the case of a 27-year-old woman who presented with chronic right knee pain and intermittent swelling for three months. Imaging revealed a subcortical osteolytic lesion in the lateral condyle of the distal femur. The patient underwent surgical curettage and biopsy, which confirmed fungal osteomyelitis caused by *Candida auris*. Antifungal susceptibility testing showed resistance to multiple agents, with sensitivity to voriconazole. Following surgical debridement, the patient was treated with prolonged oral voriconazole for six months. At follow-up, she demonstrated complete clinical and radiological recovery with a good functional outcome. This case highlights the importance of considering fungal infection in persistent osteolytic lesions and emphasizes the role of biopsy, culture, and targeted antifungal therapy in achieving successful outcomes. *C. auris* osteomyelitis is exceedingly rare, with only a limited number of cases reported in the literature.

## Introduction

Fungal osteomyelitis is an uncommon but increasingly recognized form of bone infection, characterized by its indolent onset, diagnostic challenges, and potential for significant morbidity if not treated promptly [1,2]. Unlike bacterial osteomyelitis, fungal infections of bone are rare and often occur in immunocompromised individuals, although cases in immunocompetent patients have also been reported [2,3]. The condition is most frequently caused by opportunistic fungi such as *Candida, Aspergillus*, and endemic species, depending on geographic location [3,4]. Distal femur involvement is particularly rare, making diagnosis even more difficult due to its atypical presentation and overlap with more common conditions such as bacterial osteomyelitis, malignancy, or chronic inflammatory disorders [1,3].

The pathogenesis of fungal osteomyelitis typically involves hematogenous spread, direct inoculation following trauma or surgery, or contiguous spread from adjacent infected tissues [4]. Risk factors include prolonged antibiotic use, diabetes mellitus, immunosuppressive therapy, malignancy, and previous orthopedic interventions [4]. However, in some cases, no clear predisposing factor is identified, further complicating clinical suspicion [3]. Patients often present with nonspecific symptoms such as localized pain, swelling, low-grade fever, and reduced joint mobility, which can persist for weeks or months before diagnosis [2,3].

Radiological findings in fungal osteomyelitis are variable and may mimic other bone pathologies. Imaging modalities such as X-ray, MRI, and CT scans can reveal lytic lesions, cortical destruction, or periosteal reaction, but these findings are not pathognomonic [3]. Definitive diagnosis relies on microbiological and histopathological confirmation through biopsy and culture, which is crucial for identifying the causative organism and guiding antifungal therapy [2,3].

The management of fungal osteomyelitis is often complex and requires a combination of prolonged antifungal therapy and surgical intervention [1,3]. Antifungal treatment is typically tailored based on the specific organism and its susceptibility profile, with agents such as amphotericin B, azoles, or echinocandins commonly used [2,5]. Surgical debridement plays a vital role in removing necrotic tissue, reducing fungal load, and promoting healing [3]. Delayed diagnosis or inadequate treatment can lead to chronic infection, bone destruction, and functional impairment [1,3].

This case report highlights a rare presentation of fungal osteomyelitis involving the distal femur, emphasizing the importance of maintaining a high index of suspicion, especially in patients with persistent symptoms unresponsive to conventional antibiotic therapy. Early diagnosis through appropriate imaging and microbiological evaluation, combined with timely medical and surgical management, is essential to improve patient outcomes and prevent long-term complications.

## Case presentation

A 27-year-old female patient came to the outpatient department (OPD) with complaints of deep-seated right knee pain for the past three months, which was insidious in onset, progressive in nature, aggravated on sitting, squatting, and relieved on rest. It was also associated with swelling for the past three months, which was on and oﬀ.

She gave the history of a road traﬃc accident (two-wheeler vs pedestrian) two years back, sustaining a closed injury to her right knee. She was able to bear weights and walk after the injury. She had no history of fever, weight loss, loss of appetite, or cough. She was a recently diagnosed diabetic and was on oral hypoglycemics. She was not on any treatment for any other medical ailments. She also had no other previous surgical history.

Her laboratory parameters were erythrocyte sedimentation rate (ESR) 39 mm/hr (reference range: 4-10 mm/hr), CRP 0.9 mg/dl (reference range: 0-0.8 mg/dl), and white blood cell count 10350 cells/mm^3 ^(reference range: 4000-11000 cells/mm^3^).

On examination, mild swelling was present over the knee joint. No scars, sinuses, dilated veins, or obvious muscle wasting were present. There was no obvious limb length discrepancy, and the popliteal fossa was normal without any swelling or pulsations. On palpation, there was no warmth; tenderness was present over the lateral femoral condyle. The range of movements in the right knee joint was five to 40 degrees, with fixed flexion deformity of five degrees and restricted further with pain. There was no palpable synovial thickening, no patellar tenderness, and no coronal or sagittal plane instability. The distal neurovascular status of the right lower limb was assessed and found to be normal. There were no palpable inguinal or pelvic lymph nodes.

**Figure 1:**
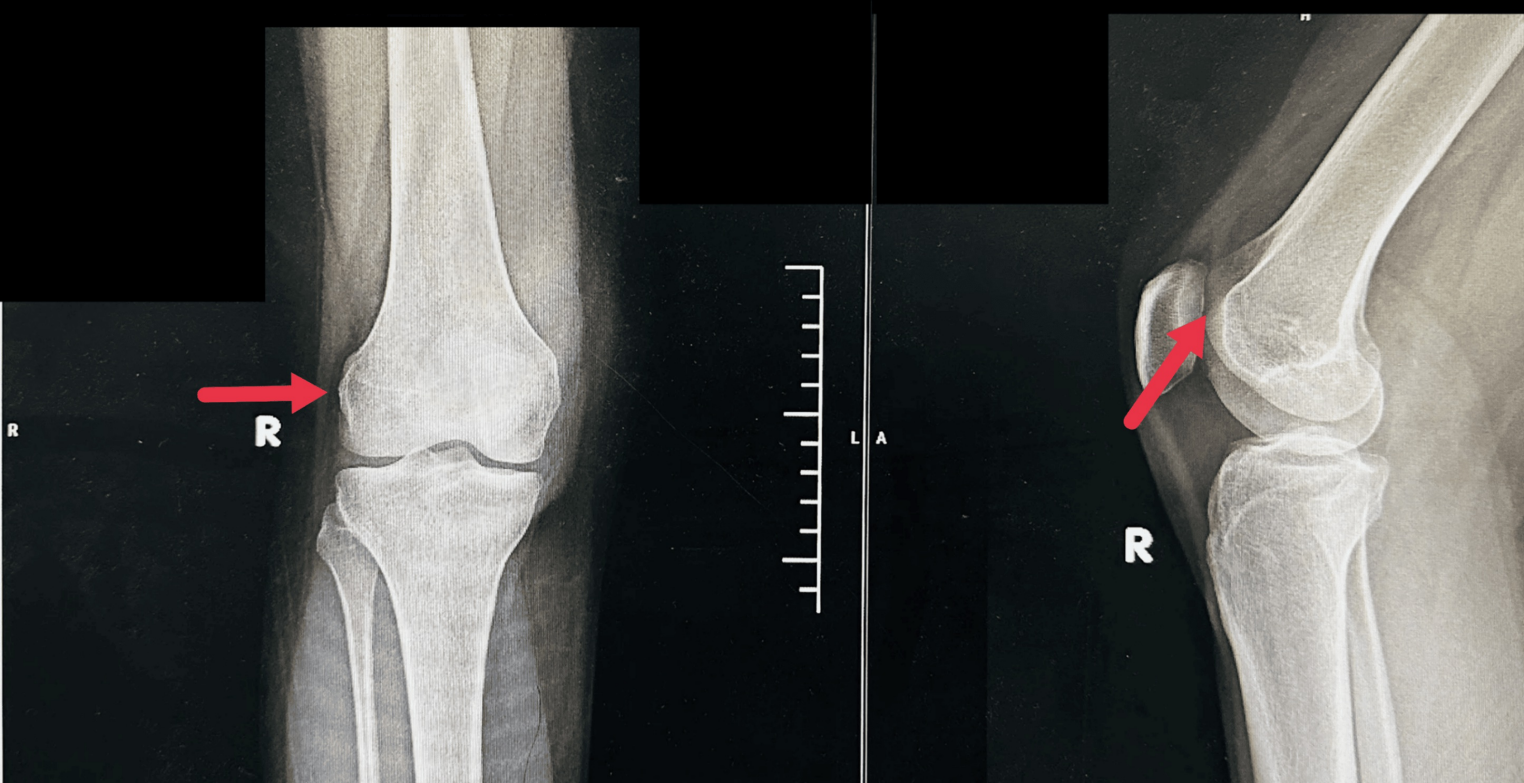
Preoperative X-ray of the right knee The red arrow shows a doubtful lytic lesion on the lateral distal femoral condyle.

Radiograph of the right knee was done, which showed a doubtful lytic lesion on the lateral distal femoral condyle (Figure 1).

**Figure 2:**
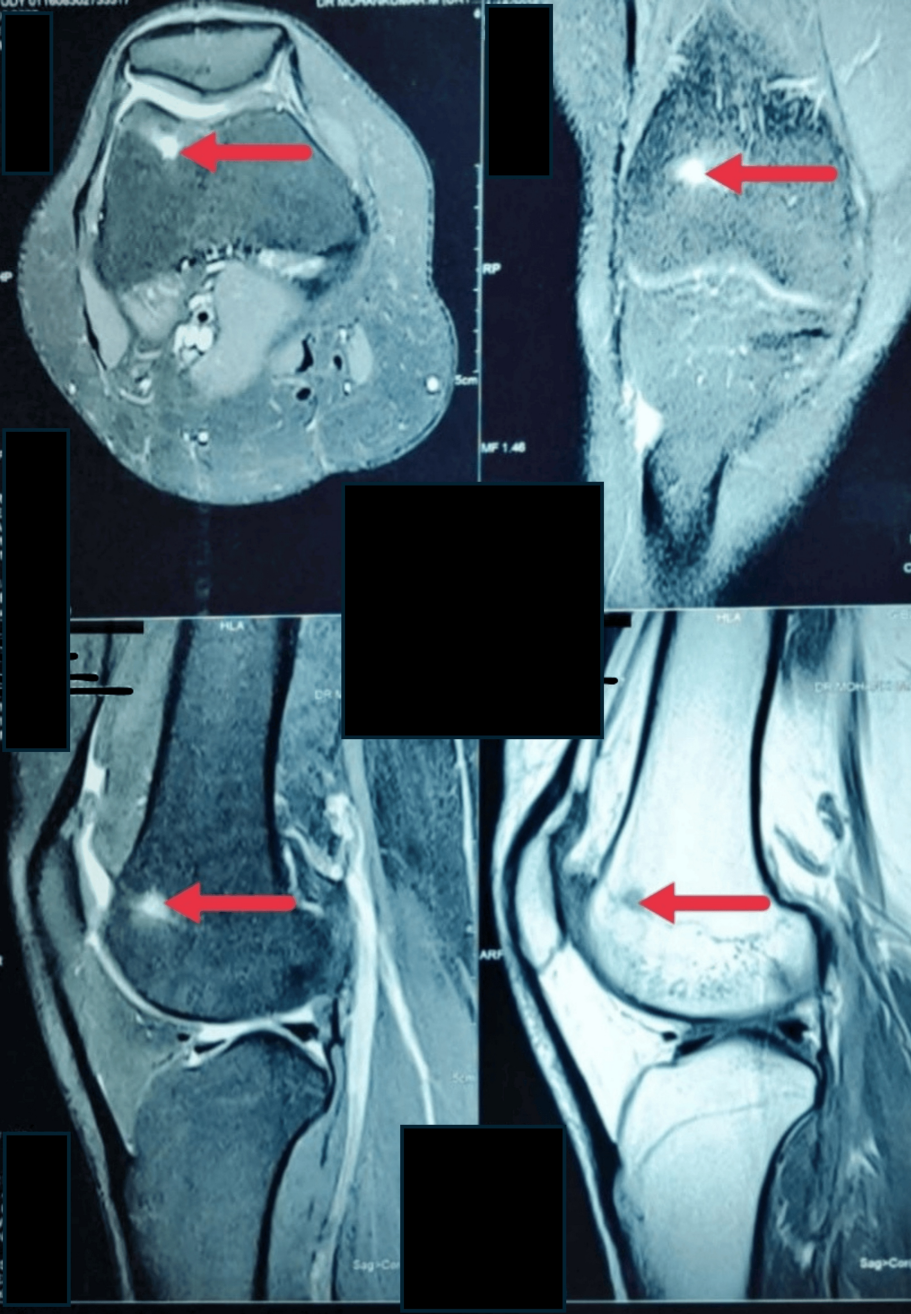
MRI of the right knee The red arrow shows a T2 hyperintense and T1 iso to hypointense lesion in the lateral distal femoral condyle.

Hence, higher imaging in the form of MRI was done and showed hypointense to isointense on T1-weighted images and hyperintense on T2-weighted/Short tau inversion recovery (STIR) sequences, suggestive of inflammatory or infective pathology. Surrounding marrow edema was noted in the adjacent distal femoral metaphysis. Mild cortical thinning with focal cortical breach was present along the lateral cortex without significant periosteal reaction. Minimal adjacent soft tissue edema was seen without a well-formed abscess or significant soft tissue extension. The dimensions of the lesion and the distance from the lateral, inferior, and anterior cortex were discerned from the images (Figure 2).

Although the radiological investigations were suggestive of osteomyelitis, a definitive diagnosis could not be achieved, and hence, she was planned for right distal femur lesion biopsy.

**Figure 3:**
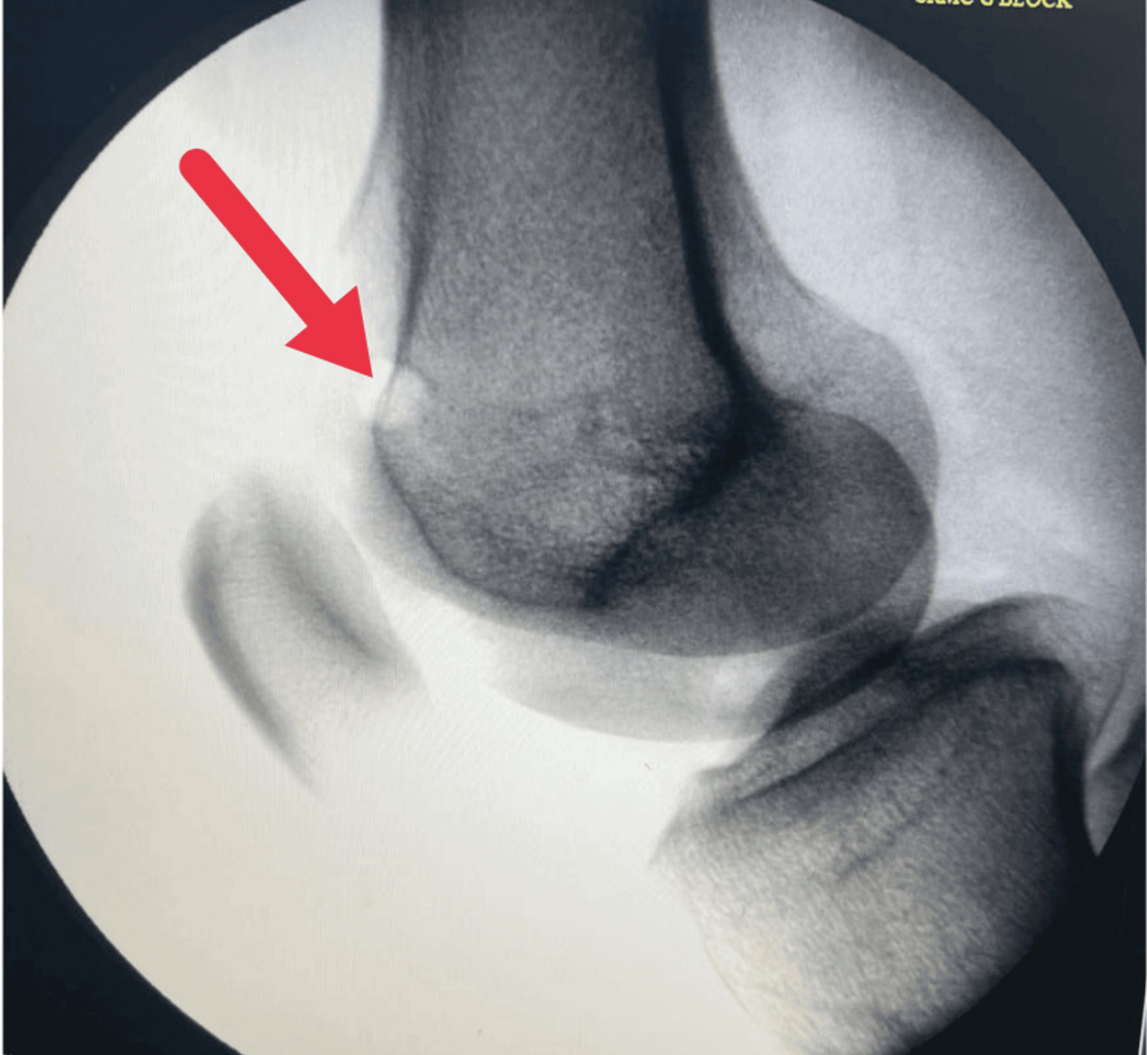
Intraoperative fluoroscopy image The red arrow shows the drilled lateral cortex, using a high speed burr.

Under pneumatic tourniquet control, under spinal anesthesia, the patient in supine position, the right lower limb parts were painted and draped. An Incision was made over the lateral aspect of the distal thigh, and the vastus lateralis was retracted upwards. The lateral condyle of the distal femur was visualized. Under fluoroscopic guidance, drilling of the cortex drilled using high speed burr with the already adjudged distances (as per the MRI) of 6 mm from anterior cortex, 20 mm from the joint line, and 12 mm from the lateral cortex. The lesion was entered and confirmed under fluoroscopy (Figure 3).

**Figure 4:**
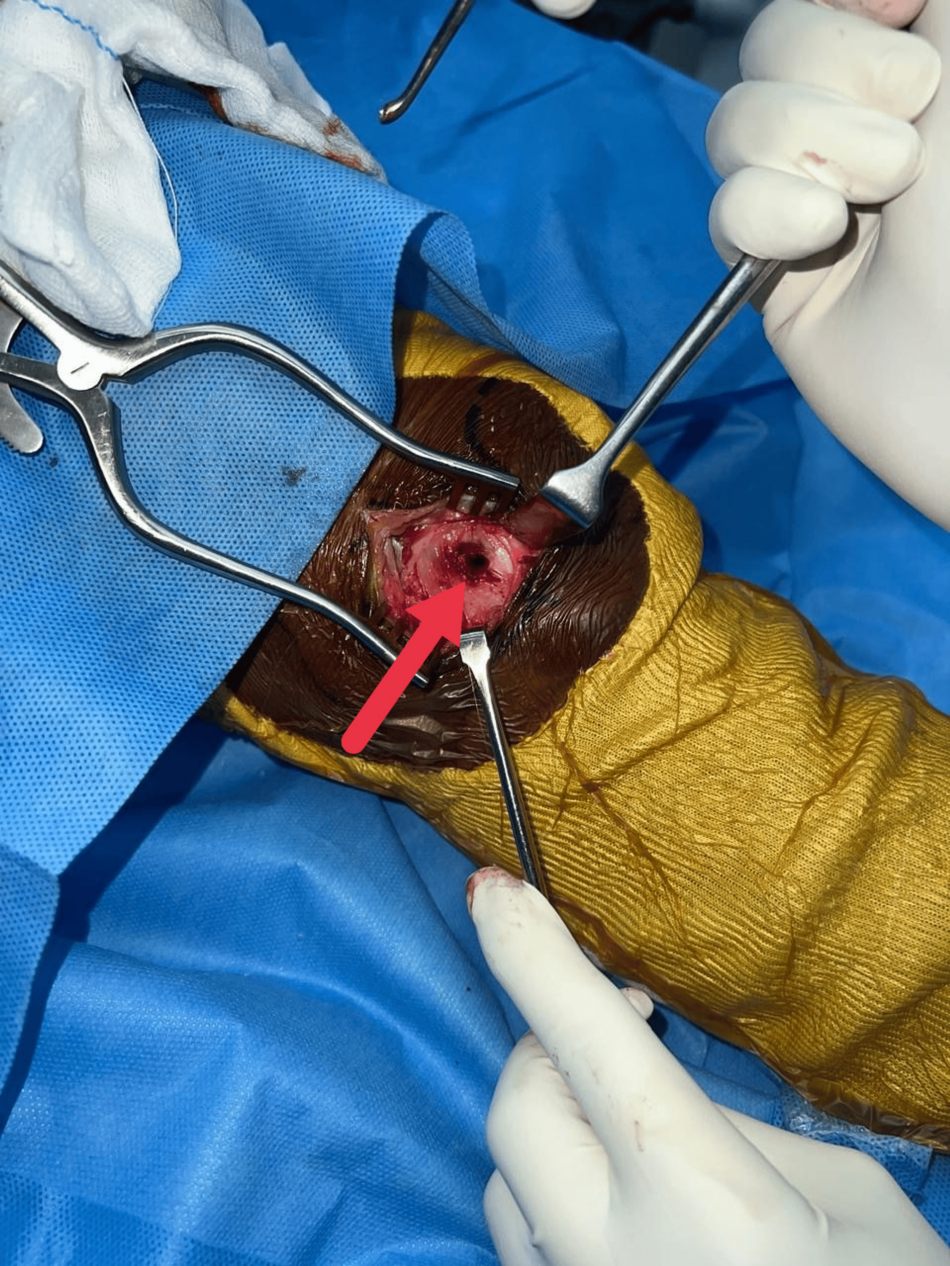
Intraoperative image showing curettage of the lesion The red arrow shows curettage of the lesion.

The contents of the lesion were soft in consistency, and through the tunnel created by drilling with the burr, the contents were curetted and sent for biopsy and culture. After thorough curettage of the lesion, the integrity of all four walls of the lesion was checked and found to be hard, comparable to a normal bone (Figure 4). 

**Figure 5:**
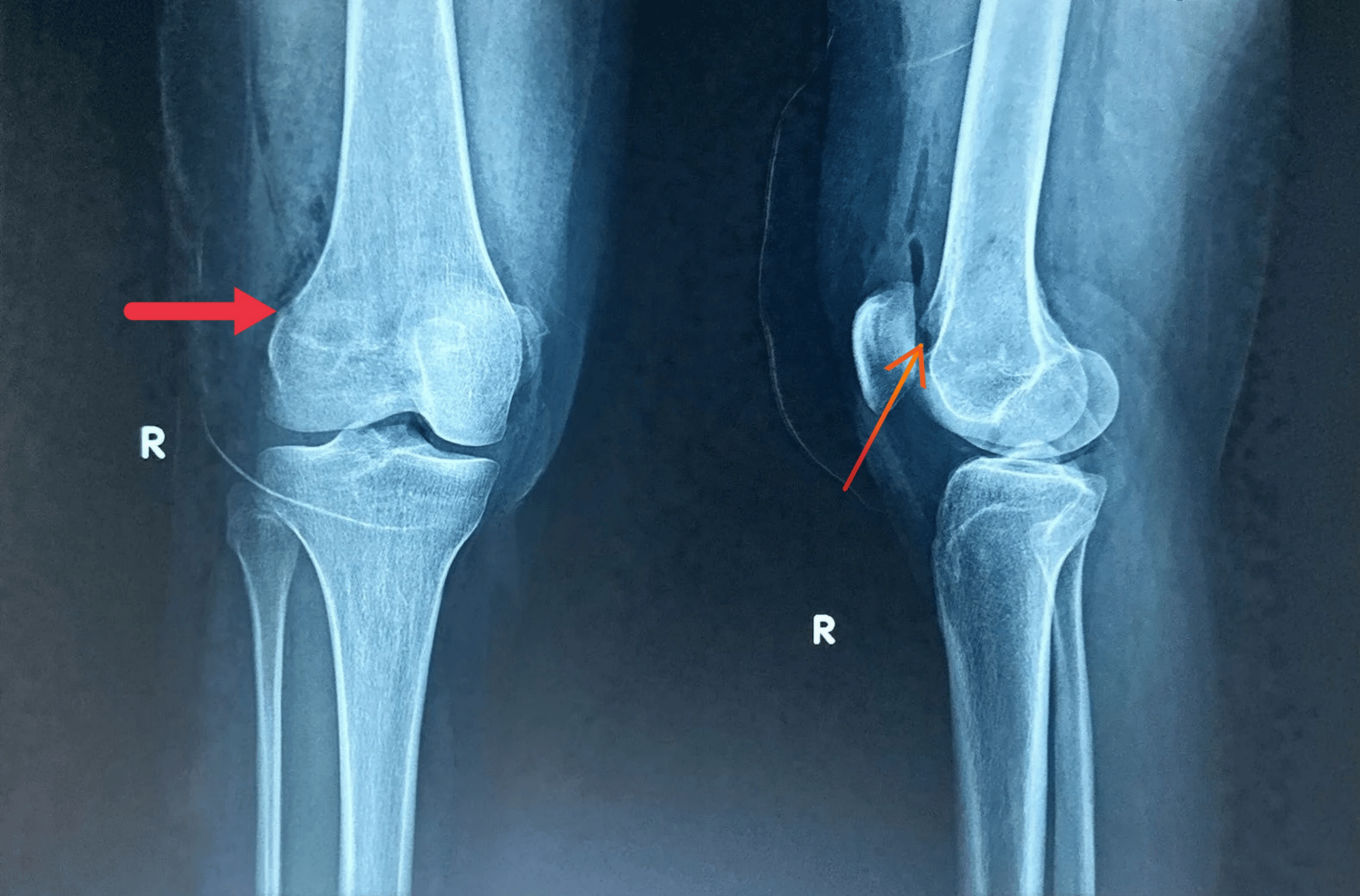
Postoperative X-ray of the right knee The red arrow shows adequate curettage of the lesion postoperatively.

A postoperative radiograph confirmed adequate curettage of the lesion (Figure 5).

On postoperative day one, the patient was started on partial weight-bearing mobilisation.

The culture showed the growth of *Candida auris* and resistance to antifungal drugs like amphotericin-B, fluconazole, and Itraconazole. Following discussion with microbiologists, she was started on voriconazole 400 mg BD orally for the first two days, followed by a maintenance dose of 200 mg BD orally for five to six months. Histopathological examination showed features suggestive of the chronic inflammatory process.

Her wound healed uneventfully, and she was discharged with oral voriconazole. She was reviewed one month and three months after the operation, and at the recent six-month postoperative period after completing her oral antifungal course, she showed excellent clinical recovery with a full range of movement of the knee from 0 to 130 degrees, with no localized tenderness at the lateral distal femoral condyle. Her quality-of-life indicators showed good functional outcomes post-procedure and after completion of the full antifungal treatment course.

## Discussion

Osteomyelitis is characterized by progressive inflammatory destruction of bone, accompanied by new bone formation, and is commonly associated with predisposing factors such as trauma, a high microbial load, or the presence of foreign material [1]. While bacterial causes are more frequently encountered, fungal osteomyelitis remains an uncommon entity and often presents with an indolent clinical course, contributing to delays in diagnosis and management. It should be strongly considered in patients presenting with persistent musculoskeletal symptoms that do not respond to conventional antibacterial therapy [2,3].

Several risk factors have been implicated in the development of fungal osteomyelitis, including diabetes mellitus, malignancy, immunosuppression, prolonged antibiotic use, and invasive procedures [4]. In the present case, the patient’s diabetic status may have contributed to increased susceptibility to infection. However, fungal osteomyelitis can occasionally occur in patients without clear predisposing factors, further complicating early clinical suspicion.

*C. auris*, first described in 2009, has emerged as a multidrug-resistant fungal pathogen of significant global concern [5,6]. One of the major challenges associated with this organism is its frequent misidentification as closely related species such as *C. haemulonii*, leading to underreporting and delays in appropriate treatment [7,8]. Invasive infections caused by *C. auris* are associated with high morbidity and mortality, with reported mortality rates ranging from 40% to 60% [9]. Additionally, epidemiological studies have highlighted its ability to cause healthcare-associated outbreaks due to persistent colonization and transmission within hospital settings [10].

The management of *C. auris* infections is further complicated by its resistance to multiple antifungal agents and the lack of well-defined susceptibility breakpoints, posing significant therapeutic challenges [11]. There are very few reported cases of *C. auris* causing osteomyelitis, and consequently, evidence guiding optimal management strategies remains limited [12]. This underscores the importance of individualized treatment planning based on microbiological culture and antifungal susceptibility testing.

Diagnosis of fungal osteomyelitis is often challenging, as clinical and radiological findings are nonspecific and blood cultures may frequently be negative. Definitive diagnosis relies on tissue biopsy with microbiological culture and histopathological examination, which remain the gold standard for identifying the causative organism. Early and accurate diagnosis is crucial in initiating appropriate targeted therapy and improving outcomes.

Adequate surgical debridement remains a cornerstone in the management of chronic osteomyelitis, as it helps in removing necrotic tissue, reducing microbial load, and enhancing the effectiveness of antifungal therapy. However, recurrence rates have been reported even after appropriate treatment, emphasizing the need for prolonged and carefully monitored therapy [13,14]. In the present case, early surgical intervention combined with targeted antifungal therapy using voriconazole resulted in an excellent clinical and functional outcome.

Strict infection control measures are essential to prevent nosocomial transmission of *C. auris*, particularly in healthcare settings where outbreaks have been documented. Effective surveillance, adherence to infection control protocols, and appropriate antifungal stewardship are critical in limiting the spread of this organism [15].

This case highlights the importance of considering fungal osteomyelitis in the differential diagnosis of chronic bone lesions, especially in patients with underlying risk factors such as diabetes mellitus or in those who do not respond to standard antibacterial therapy. A high index of suspicion, combined with timely microbiological evaluation and appropriate medical as well as surgical management, is essential to achieve favorable outcomes.

## Conclusions

We report a case of fungal osteomyelitis in the distal femur caused by an infection with *C. auris*. This case highlights the importance of higher imaging, the role of intraoperative fungal cultures to be included with routine bacterial cultures, early initiation of antifungals, and the importance of keeping fungal osteomyelitis as a potential differential diagnosis. This was the inaugural instance of *C. auris* osteomyelitis observed at our institution, with no contemporaneous cases reported. Prompt diagnosis, multidisciplinary management, and prolonged targeted antifungal therapy were crucial in achieving favorable clinical and functional recovery in this patient.
